# Close of the Session

**Published:** 1857-09

**Authors:** 


### Close of the Session.

The third session of the Atlanta Medical College will close on the 3d of September, with 124 Matriculates, a very respectable increase upon the *number* of the last session, but we have no hesitation in saying that in this has been much the smallest advance in the standing and influence of the institution. The high object which has been continually held in view by the Faculty from the commencement, to furnish facilities of the first order, and to build up an institution inferior to none, is beginning to be realized and extensively acknowledged.

While the Faculty have submitted to great sacrifices to accomplish this success which, they have no hesitation in asserting, has been of greater benefit to others than themselves, they only ask the *most simple justice* in the way of a fostering interest upon the part of the city and the State in which the institution is located, to enable them to present advantages which will command a patronage from every Southern State, some six or seven of which are represented in the present class.

